# Structure prediction of magnetosome-associated proteins

**DOI:** 10.3389/fmicb.2014.00009

**Published:** 2014-01-29

**Authors:** Hila Nudelman, Raz Zarivach

**Affiliations:** ^1^Department of Life Sciences, Ben-Gurion University of the NegevBeer Sheva, Israel; ^2^National Institute for Biotechnology in the Negev, Ben-Gurion University of the NegevBeer Sheva, Israel

**Keywords:** magnetosome, structure prediction, Protein structure–function, magnetotactic bacteria, membrane invagination

## Abstract

Magnetotactic bacteria (MTB) are Gram-negative bacteria that can navigate along geomagnetic fields. This ability is a result of a unique intracellular organelle, the magnetosome. These organelles are composed of membrane-enclosed magnetite (Fe_3_O_4_) or greigite (Fe_3_S_4_) crystals ordered into chains along the cell. Magnetosome formation, assembly, and magnetic nano-crystal biomineralization are controlled by magnetosome-associated proteins (MAPs). Most MAP-encoding genes are located in a conserved genomic region – the magnetosome island (MAI). The MAI appears to be conserved in all MTB that were analyzed so far, although the MAI size and organization differs between species. It was shown that MAI deletion leads to a non-magnetic phenotype, further highlighting its important role in magnetosome formation. Today, about 28 proteins are known to be involved in magnetosome formation, but the structures and functions of most MAPs are unknown. To reveal the structure–function relationship of MAPs we used bioinformatics tools in order to build homology models as a way to understand their possible role in magnetosome formation. Here we present a predicted 3D structural models’ overview for all known *Magnetospirillum gryphiswaldense* strain MSR-1 MAPs.

## INTRODUCTION

Magnetotactic bacteria (MTB) are a group of Gram-negative aquatic prokaryotes that can synthesize a unique prokaryotic organelle, called a magnetosome (Bazylinski et al., 2004). The magnetosome contains magnetic crystals enclosed within membrane vesicles, which are aligned as intracellular chains along the cell (Balkwill et al., 1980; Komeili et al., 2006). The magnetosome membrane creates an isolated environment in the cell which is important for mineral crystal nucleation and growth (Komeili et al., 2004). The magnetosome chain forces the bacteria to align passively to the geomagnetic field and the bacteria then swim accordingly with the use of their flagella, a behavior called magnetotaxis (Balkwill et al., 1980; Lower and Bazylinski, 2013). Magnetotaxis is believed to aid MTB to reach regions of optimal oxygen concentrations without long, random movements (Gorby et al., 1988).

The magnetic crystals consist of magnetite (Fe_3_O_4_) or greigite (Fe_3_S_4_). Their size typically ranges between 35 and 120 nm and are in the size range of a single-magnetic-domain (SD; Frankel et al., 1979; Mann et al., 1990; Kirschvink, 1992). Each bacteria has a defined species-dependent crystal size and shape and contains one or more magnetosome chains (Balkwill et al., 1980; Bazylinski and Frankel, 2004). The consistent morphology in different species or strains of the magnetic crystals indicates that mineral formation is a highly controlled process (Frankel and Bazylinski, 2009).

It was found that magnetosome formation is under strict control of specific gene expression (Schübbe et al., 2003). Most of these genes are located on a genomically conserved region, known as the magnetosome island (MAI; Schübbe et al., 2003). The MAI is found to be highly conserved in almost all MTB species and includes a highly conserved and essential operon (*mamAB*), as well as three less conserved operons (*mamGFCD, mms6, and mamXY*; Fukuda et al., 2006). It was shown that MAI or *mamAB* operon deletion causes the loss of magnetosome formation (Murat et al., 2010; Lohsse et al., 2011).

MamAB operon in *M. gryphiswaldense* MSR-1 includes 17 open reading frames which correspond to ~16.4 kb of DNA (Schübbe et al., 2006). This operon contains genes that are essential for magnetosome formation and have important functions such as membrane invagination, iron transport, and magnetite biomineralization (Lohsse et al., 2011). The *mamAB* cluster encodes proteins that are essential for membrane invagination and magnetosome biogenesis (*mamB, E, I, L,* and *Q*), magnetosomal iron transport (*mamB* and *M*), magnetite biomineralization (*mamE, O, T, P,* and *S*), and magnetosome chain assembly (*mamK* and *mamJ*; Murat et al., 2010; Yang et al., 2010; Quinlan et al., 2011; Uebe et al., 2011).

In *M. gryphiswaldense* MSR-1, the *mamXY* operon is a ~4.9 kb segment which is located ~28 kb downstream of the *mamAB* operon and consists of *mamY*, *mamX*, *mamZ* (*mamH*-like), and *ftsZ-*like genes (Ding et al., 2010). *mamXY* is conserved among all *Magnetospirillum* bacteria. It was shown in both *M. gryphiswaldense* MSR-1 and *M. magneticum* AMB-1 that *mamXY*-encoding proteins are associated with the magnetosome membrane (Lohsse et al., 2011). Deletion of *M. gryphiswaldense* MSR-1 *mamXY* causes short magnetosome chains with regular shape but smaller particles (Lohsse et al., 2011).

The *mamCD* (2.1 kb) and *mms6* (3.6 kb) operons are not essential for biomineralization but they encode genes which control the size and shape of magnetite particles (Scheffel et al., 2008; Murat et al., 2010; Lohsse et al., 2011; Tanaka et al., 2011; Murat et al., 2012). Deletion of the *mamCD* operon – which contains four genes (*mamC*, *mamD*, *mamF,* and *mamG*) – results in crystals with approximately 75% of the wild type size (Scheffel et al., 2008). The *mms6* operon is located upstream of the *mamCD* operon and contains five genes (*mms6*, *mmsf*, *mgr4070*, *mgr4071,* and *mgr4074*; Faivre and Schüler, 2008). Co-deletion of both operons results in further reduction in the shape regularity and alignment of magnetite crystals (Lohsse et al., 2011).

Based on current scientific data it is suggested that magnetosome formation occurs via several steps that can act simultaneously. These include, (i) protein sorting and inner membrane invagination, (ii) alignment of the magnetosome into chains, (iii) iron uptake and crystal nucleation, and (iv) crystal maturation (Murat et al., 2010). Today, a general model for magnetosome formation and protein involvement is based on genetic approaches but most of the protein functions have yet to be determined (Murat et al., 2010).

One of the most studied strains is the magnetotactic α-proteobacterium *Magnetospirillum gryphiswaldense* MSR-1, which contains all four magnetosome operons (Lohsse et al., 2011). *M. gryphiswaldense* MSR-1 creates more than 60 cubo-octahedral magnetite-containing magnetosomes (Schleifer et al., 1991). In *M. gryphiswaldense* MSR-1 there are over 28 different proteins that are involved in magnetosome formation and most of the corresponding genes are located on the MAI (Schübbe et al., 2006; Richter et al., 2007). Some of these proteins are homologous to known protein families, such as: tetratricopeptide repeat (TPR) proteins, CDF transporters, PDZ proteins, proteases, and more (Okuda et al., 1996).

In order to understand the function of each protein during magnetosome formation it is essential to find their structure–function relationships. Nowadays there are only a few known MTB protein structures that promote understanding of the protein roles during magnetosome formation. MamA and MamP are the only proteins from the *mamAB* operon whose 3D structures have been determined (Zeytuni et al., 2011, 2012; Siponen et al., 2013). In this work we predicted and analyzed the 3D models of magnetosome proteins from *M. gryphiswaldense* MSR-1 strain as a way of elucidating their functions during the processes of magnetosome formation and present them based on their main predicted function or their association with protein families.

## PROTEIN SORTING AND MAGNETOSOME MEMBRANE INVAGINATION

### MamI

**FIGURE 1:**
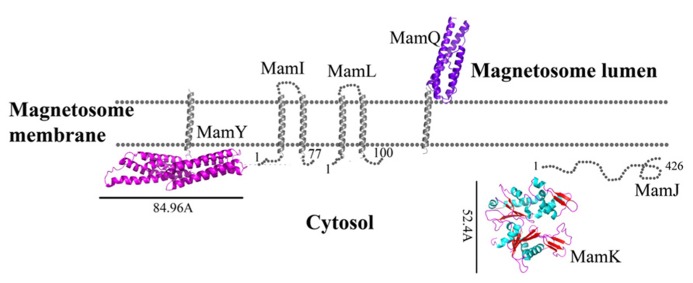
**Protein structure predictions that are involved in protein sorting, magnetosome membrane invagination, and magnetosome chain assembly.** Structures are in ribbon representation. Predictions of anchoring transmembrane helices are in gray. Black bars define the protein size in angstroms. *M. gryphiswaldense* MSR-1 MamA structure was determent by X-ray crystallography.

MamI is a small, 77 residue protein with two predicted integral membrane α-helices (Komeili, 2012). MamI deletion results in the loss of the magnetosome membrane, which might be indicative of MamI involvement in magnetosome membrane invagination (Komeili, 2012). MamI-GFP was shown to be localized to the magnetosome and indicates the presence and position of the magnetosome in the cell (Murat et al., 2010). MamI has no homologous domains or proteins except for its homologues in other MTB species. MamI secondary structure prediction yields a short loop of three amino acids (Thr35, Glu36, and Leu37) between the transmembrane helices (**Figure 1**; Slabinski et al., 2007). This loop is not predicted to interact with the magnetic particle, which may indicate that MamI has the ability to bend the magnetosome membrane.

### MamQ

MamQ deletion in AMB-1 cells results in the complete loss of magnetosome formation (Murat et al., 2010). MamQ is predicted to be an integral membrane protein with 273 amino acids and is homologous to the LemA protein family (Greene and Komeili, 2012). LemA proteins are predicted to have a membrane-spanning domain on their N-terminal in which the non-membrane C-terminal domain (CTD) will be extracellular (pointing towards the magnetosome lumen); yet, its role is still unknown (Lenz et al., 1996).

**FIGURE 2:**
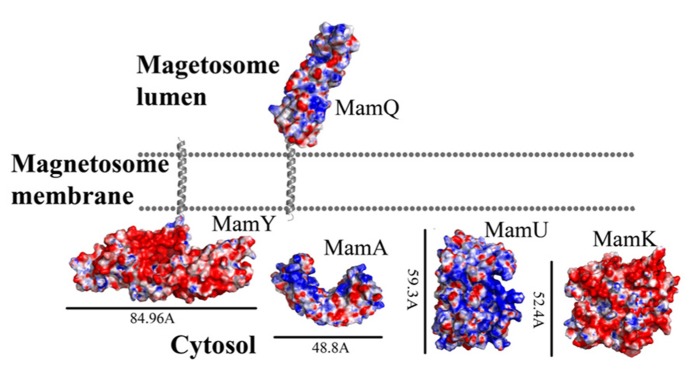
**Electrostatic density representation of proteins involved in protein sorting, magnetosome membrane invagination, and magnetosome chain assembly.** Negative charges are in red and positive charges are in blue. Predictions of anchoring transmembrane helices are in gray. Black bars define the protein size in angstroms. *M. gryphiswaldense* MSR-1 MamA structure was determent by X-ray crystallography.

MamQ secondary structure prediction using XtalPred and PsiPred servers show that MamQ contains a cytosolic unstructured N-terminal followed by an integral membrane helix and a CTD with a helix-turn-helix fold (**Figure 1**; Slabinski et al., 2007). The model structure of MamQ was based on the LemA template (PDB ID: 2ETD; Arnold et al., 2006). The model presents only the cytosolic-terminal domain (Asn70 to Thr223; **Figure 1**). Multiple sequence alignment shows high similarities between MamQ proteins from other MTB species and good conservation to important residues in *Listeria monocytogenes* LemA (Li, 2003). The MamQ model presents a magnetosomal domain with a negatively charged surface similar to the LemA structure, whose function is still unknown (**Figure 2**).

### MamL

The *mamL* gene is located within the *mamAB* operon and its deletion results in the loss of the magnetosome membrane in *M. magneticum* AMB-1 (Murat et al., 2010). MamL is a small, 123 amino acid protein containing two predicted integral membrane α-helices and is very similar to MamI (**Figure 1**; Komeili, 2012). MamL is found only in MTB species and there are no homologous proteins or domains in other organisms (Komeili, 2012).

### MamA

MamA is one of the most conserved MAPs and exists in all MTB MAI (Zeytuni et al., 2011). MamA deletion does not have an effect on membrane invagination or iron oxide biomineralization (Komeili et al., 2004; Zeytuni et al., 2011). It was shown that MamA localization is dynamic during cell growth and is not dependent on active magnetite formation (Komeili et al., 2004). It was also shown that MamA-GFP expressed in *M. magneticum* AMB-1 cells were localized to the magnetosome chain and surrounded the magnetosome vesicles (Yamamoto et al., 2010; Zeytuni et al., 2011).

MamA contains several TPR domains and self-assembles into stable homo-oligomeric complexes (Okuda et al., 1996; Zeytuni et al., 2011). TPR motifs are known to be involved in protein–protein interactions and are present in a number of proteins that are functionally unrelated (Blatch and Lässle, 1999). TPR is a 34 amino acid structure arranged as repeats of antiparallel α-helices (Das et al., 1998).

It was shown that purified MamA forms round-shaped complexes with a central pore cavity (Zeytuni et al., 2011). Mutation in the first helix of the MamA TPR motif causes disassembly of MamA complexes, which indicates its involvement in complex formation (Zeytuni et al., 2011). The conserved salt bridge between Arg50-Asp79 in *M. magneticum* AMB-1 is responsible for stabilization of the N-terminal domain, which is important to MamA complex formation and localization to the magnetosome chain (Zeytuni et al., 2011). The structures of the MamA deletion mutant from *M. magneticum* AMB-1, *M. gryphiswaldense* MSR-1, and *Magnetobacterium bavaricum* are composed of 10 antiparallel α-helices that are folded as five TPR motifs and form a hook-shaped structure (PDB ID: 3AS4, 3AS8, and 2MUC; **Figure 1**; Zeytuni et al., 2011). It was suggested that MamA has three binding sites, two which are needed to create the round homo-oligomeric complexes and one to interact with the magnetosome chain (Zeytuni et al., 2011). Based on many TPR-ligand structures it was shown that the TPR protein family can bind unstructured peptides, helices, or entire TPR motifs (Zeytuni et al., 2011). Compared to other TPR proteins, which have a positive or negative binding pocket in their concave surface, MamA from *M. magneticum* AMB-1 displays a highly positive binding site in the concave surface similar to other MTB species (**Figure 2**; Zeytuni et al., 2011, 2012). The convex surface charge in *Magnetospirillum* species is negative, unlike in *M. bavaricum* strain that presents both positive and negative patches (Zeytuni et al., 2012). It was predicted that the MamA convex side may act as a binding site with other magnetosome-associated proteins (MAPs; Zeytuni et al., 2011). MamA was found to interact with four different proteins or protein fragments *in vivo* (of 26.8, 31.6, 54, and 63.5 kDa) which supports the suggested model in which the MamA convex surface faces the magnetosome membrane and acts as a multi-protein assembly site (Yamamoto et al., 2010; Zeytuni et al., 2011).

### MamU

MamU is a 297 amino acid protein predicted to fold as a mixed α-helices-β-sheets structure (Slabinski et al., 2007). Δ*mamU* mutants did not yield any changes in their magnetic response (Murat et al., 2010). One MamU homologous protein family are the diacylglycerol kinases (DGKs), which phosphorylate the second-messenger diacylglycerol (DAG) to phosphatidic acid (PA; Van Blitterswijk and Houssa, 2000). The DGK pathway is known to be a major player in the regulation of cell response (Mérida et al., 2008). Nowadays there are nine known members of the DGK family which contain the conserved catalytic domains and two cysteine rich domains on the protein N-terminal (Van Blitterswijk and Houssa, 2000). The catalytic domain is known to have six conserved aspartate residues which play a major role in the enzyme’s activity (Van Blitterswijk and Houssa, 2000).

The MamU model structure is based on a DGK structure (ID: 3T5P chain L; **Figure 1**; Guex and Peitsch, 1997). Sequence alignment between MamU and DGK shows that the conserved aspartates do not appear in the MamU sequence and there is only one cysteine residue on the MamU N-terminal domain. Changes in the electrostatic density map between MamU and DGK indicate that MamU has a different substrate and activity, or that the fold of MamU is different from the DGK protein family.

### MamY

MamY is a 371 amino acid protein predicted to have two integral membrane helices at its N-terminal and a large cytosolic domain at the C-terminal (Slabinski et al., 2007). MamY has a weak homology to BAR domain proteins, which are known to be involved in cellular membrane dynamics (Peter et al., 2004; Tanaka et al., 2010). Deletion of the *mamY* gene yielded an enlarged magnetosome vesicle phenotype with small magnetite crystals (Tanaka et al., 2010). In *M. magneticum* AMB-1 MamY-GFP were localized near to the magnetosome vesicles with small magnetite crystals that are still attached to the inner membrane (Tanaka et al., 2010). It was suggested that MamY’s role is to constrict the magnetosome membrane during its invagination, followed by magnetite crystal growth (Tanaka et al., 2010).

While searching for MamY structural models, several proteins were suggested as templates (Arnold et al., 2006; Kelley and Sternberg, 2009). One of these templates was the cytoplasmic domain of a bacterial chemoreceptor from *Thermotoga maritime*, which has a structure of two antiparallel helices that dimerize into a four-helix bundle with another methyl-accepting chemotaxis protein (MCP) subunit (Park et al., 2006). MCP is a transmembrane kinase which is involved in the signaling network that controls bacterial chemotaxis (Park et al., 2006). In addition, MamY structural analysis using Phyre2 shows low conservation to the domain from talin protein (PDB ID: 1SJ8; Kelley and Sternberg, 2009). Talin is a cytoskeletal protein that is known as a linker between actin and the membrane via integrin proteins, which are involved in cell adhesions (Niggli et al., 1994). The talin domain is composed of an N-terminal five-helix bundle and a C-terminal four-helix bundle, which are connected by a short loop (Niggli et al., 1994). MamY model structure shows the cytosolic domain, which is composed of four α-helices on its N-terminal followed by five α-helices (**Figure 1**). The electrostatic density map of the MamY model presents a highly negatively charged surface, similar to talin’s surface charge distribution (**Figure 2**; Papagrigoriou et al., 2004). Sequence alignment between MamY and talin presents low identity, whilst the MamY model structure has high confidence (95.36%) with the talin structure (Kelley and Sternberg, 2009). From these results we can suggest that MamY may function similarly to the talin protein and plays a role in membrane invagination and magnetosome separation from the inner membrane (Tanaka et al., 2010).

## MAGNETOSOME ARRAGMENT INTO CHAIN STRUCTURE

### MamJ

MamJ is essential for the magnetosome chain structure and its deletion leads to a new magnetosome arrangement as 3D clusters instead of a linear chain (Scheffel and Schüler, 2007). MamJ is known as an acidic protein which contains 426 amino acids with a repetitive domain structure. It has a central acidic repetitive domain (CAR domain) which is composed of an 88 amino acid motif followed by tandemly arranged copies of a highly acidic, 20 amino acid motif, consisting primarily of Pro and Glu residues arranged in Glu-Pro (Scheffel et al., 2006; Scheffel and Schüler, 2007). The CAR domain is not required for magnetosome restoration in Δ*mamJ* cells, unlike regions at the N- or C-terminal in MamJ (Scheffel and Schüler, 2007).

It is known that MamJ is associated with MamK filaments and magnetosome vesicles, thus creating a linear chain (Scheffel et al., 2006). The interactions between MamJ and MamK apparently are mediated by two protein–protein interaction domains, one located on the C-terminal and the other located in the MamJ N-terminal region (Scheffel and Schüler, 2007). Recently it was shown, by using FLIM-FRET technique, that co-expression of MamK_mCherry and eGFP_MamJ in *Escherichia coli* create a stable interaction between them which is important for magnetosome alignment (Carillo et al., 2013).

Prediction of MamJ structure using IUPRED and XtalPred servers shows that most of the protein is unstructured (**Figure 1**; Dosztányi et al., 2005; Slabinski et al., 2007). Additional analysis of the MamJ sequence using the ProDom server has found two conserved domains which exist in TonB protein (Bru et al., 2005). TonB is a highly proline-rich protein that includes a segment consisting of multiple X-Pro dipeptide repeats with an anchor to the cytoplasmic membrane via a single N-terminal transmembrane helix (Hannavy et al., 1990). TonB also functions as a mechanical linkage between the inner and outer membranes through protein–protein interactions (Ollis and Postle, 2012). These predictions support the previous evidence that MamJ can interact with MamK via protein–protein interactions and is involved in magnetosome chain assembly.

### MamK

The *mamK* gene encodes a filamentous structure which is similar to prokaryotic MreB and ParM (Sonkaria et al., 2012). MreB-like proteins polymerized into helical filamentous structures that run along the cell length (Carballido-López, 2006). The 3D structure of MreB shows high similarity to eukaryotic actin. The actin-like proteins, similar to actin, function in prokaryotic cells and are required for targeting and positioning proteins or molecular complexes (Carballido-López, 2006).

In *M. magneticum* AMB-1 strain, *mamK* deletion abolishes the filamentous structure near the magnetosome (Komeili et al., 2006), whereas *ΔmamK* in *M. gryphiswaldense* MSR-1 results in shorter, fragmented, and off-center chains and was suggested to be involved in proper magnetosome chain positioning and segregation (Katzmann et al., 2010, 2011). The function of MamK is to organize the magnetosome chain along the cell axis and associate with MamJ, which may act as an anchor between MamK and the magnetosome membrane (Sonkaria et al., 2012). Previously, it was shown that the localization of MamK filaments in the cell does not depend on the presence of MamJ (Scheffel and Schüler, 2007).

Biochemical studies indicate that MamK polymerization is the result of ATP binding whilst filament disassembly happens during ATP hydrolysis (Ozyamak et al., 2013). It seems that ATP stabilizes MamK filaments and prevents their aggregation (Ozyamak et al., 2013). *M. magneticum* AMB-1 MamK form dynamic filaments *in vivo* which depend on the ATPase active site and two proteins (MamJ and LimJ) which are required for promoting MamK filament formation (Draper et al., 2011). Furthermore, MamK filament structure has an architecture of two parallel strands, unlike MreB filaments which are linked in an antiparallel arrangement (Ozyamak et al., 2013). The MamK homology model is based on MreB’s structure (PDB ID: 1JFA; Guex and Peitsch, 1997; Sonkaria et al., 2012). The MamK model structure has four domains that are similar to the conserved secondary structure of the actin-like protein family (**Figure 1**; Carballido-López, 2006; Sonkaria et al., 2012). The electrostatic potential density map of MamK and MreB also shows high similarities, especially in the ATP binding site, suggesting that MamK has a similar activity (**Figure 2**).

## IRON TRANSPORT AND NUCLEATION

### MamO

MamO is a large, 65.3 kDa protein whose deletion leads to an empty magnetosome phenotype suggesting that MamO might be involved in biomineralization, iron transport, and/or magnetite nucleation, or allows for a suitable environment for magnetite synthesis in the magnetosome (Murat et al., 2010). MamO is an integral membrane protein with eight predicted transmembrane α-helices. Based on ProDom prediction, MamO contains two domains: one is similar to the trypsin-like peptidase and the second is similar to integral membrane proteins. The latter are involved in the transport of anions across the cytoplasmic membrane during taurine metabolism as an exporter of sulfoacetate (Yang et al., 2010). Although it seems that MamO has a predicted trypsin-like peptidase domain, expression of MamO in *E. coli* does not show any protease activity. In addition, several single point mutations (T225A, H116A, and D149A) in the predicted active site of the MamO trypsin domain did not affect magnetite crystal formation (Yang et al., 2010; Quinlan et al., 2011).

**FIGURE 3:**
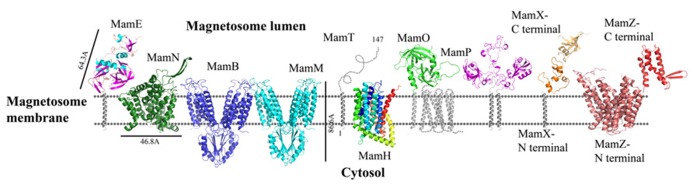
**Protein structure predictions that are involved in iron transport and nucleation.** Structures are in ribbon representation. Predictions of anchoring transmembrane helices are in gray. Black bars define the protein size in angstroms. *Magnetotactic ovoidal bacterium* MO-1 MamP structure was determent by X-ray crystallography.

Today there is no direct evidence that any part of MamO has trypsin or protease activity. To explore whether MamO can act as an active protease, we decided to build a homology model using the Swiss-Model server which can aid with understanding MamO function from a structural aspect (Arnold et al., 2006). First, we performed multiple sequence alignment between all known MamO sequences that were found in other MTB species by using the ClustalW server and ESPript 2.2^1^ (Li, 2003). This alignment indicates that all MamO variants are highly conserved and contain similar amino acids in the suspected catalytic triad. To further study the MamO fold we analyzed its 3D structure prediction from which we could determine that MSR-1 MamO shares high similarity to the trypsin-like conserved fold (**Figure 3**). The MamO modeled structure is based on the structure of two different proteins from the HtrA serine protease family (PDB ID: 1LCY, 2Z9I). The first is the mitochondrial serine protease HtrA2 from mammals and the second is HtrA2 from *Mycobacterium tuberculosis*. These proteases contain a protease catalytic triad of His, Ser, and Asp and a PDZ domain on their C-terminal. Both domains are highly conserved in the HtrA family and the PDZ domain is involved in protein–protein interactions (Pallen and Wren, 1997). The structure model of MamO does not contain the PDZ domain which is essential for the HtrA protease activity (Iwanczyk et al., 2007). In contrast to several proteases from the HtrA family, we observe that two residues – Ser and Asp – of the conserved catalytic triad do not appear in the same position in *M. gryphiswaldense* MSR-1 MamO (**Figure 3**).This, together with the lack of the PDZ domain, indicates that MamO is most likely missing the protease activity.

### MamE

MSR-1 MamE is a large, 655 amino acid protein which is important for protein localization to the magnetosome membrane and is predicted to fold as a putative serine protease with two PDZ domains and a predicted membrane anchoring the hydrophobic helix on its N-terminal (Yang et al., 2010; Siponen et al., 2012). Recently it was discovered that MamE contains a putative cytochrome c-like domain with a CXXCH motif that acts as covalent thioether bonds to the heme vinyl groups (magnetochrome; Bowman and Bren, 2008; Siponen et al., 2012). Deletion of MamE *in vivo* leads to empty magnetosome vesicles and to the loss of magnetite synthesis (Murat et al., 2010). As a putative serine protease, MamE has a highly conserved catalytic triad. It was shown that site-directed mutagenesis of these residues in *M. magneticum* AMB-1 MamE (His198, Asp221, Ser297) does not affect magnetite crystal nucleation but results in a phenotype characterized by smaller magnetite crystals and the loss of the magnetic response (Quinlan et al., 2011). The same phenotype is similar to the deletion of the magnetochrome domain (Quinlan et al., 2011). There are few hypotheses regarding the functions of magnetochrome, such as electron donation to oxidized iron, extraction of electrons to maintain the magnetosome redox state or to act as a redox buffer to maintain the balance between maghemite and magnetite (Siponen et al., 2012).

**FIGURE 4:**
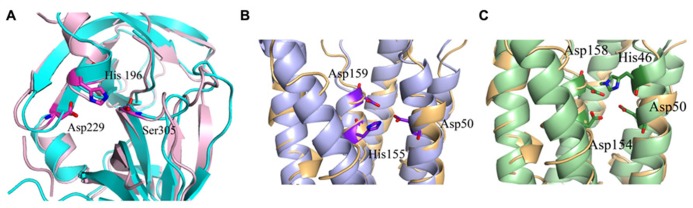
**Predicted catalytic/ binding sites of MamE, MamM, and MamB. (A)** MamE predicted structure (light pink) overlapping with serine protease structure (cyan); the catalytic triad is shown as sticks. **(B)** Overlap of the MamM predicted structure (light blue) with zinc transporter FieF (light orange); the three residues that are predicted to create the negative pocket are presented as sticks. **(C)** Overlap of the MamB predicted structure (light green) with zinc transporter FieF (light orange), the three residues that are predicted to create negative pocket are presented as sticks.

To understand MamE function and how it is involved in magnetosome formation, we decided to create a homology model. The MSR-1 MamE model was built using the Swiss-PdbViewer program and is based on a serine protease structure (PDB ID: 2Z9I; Guex and Peitsch, 1997; **Figure 3**). From the 3D structure and the multiple sequence alignment we can locate the highly conserved catalytic triad of serine proteases, yet it was not shown experimentally whether MamE has protease activity (**Figure 4**).

Based on domain prediction by InterProScan server and the available 3D model of MamE, a PDZ domain is predicted. PDZ domains are usually known to be involved in protein–protein interactions and signaling complexes (Lee and Zheng, 2010). In a previous study, it was shown that MamE deletion caused mislocalization of magnetosome proteins which could indicate the importance of the PDZ domain to protein–protein interactions (Murat et al., 2010). Prediction of a MamE PDZ domain shows high conservation to PDZ architecture, which consists of six anti-parallel β-strands and two α-helices (Sheng and Sala, 2001). Furthermore, the PDZ domain was shown to interact with the conserved PDZ binding signature at the C-terminal of MSR-1 MamB (Uebe et al., 2011). The major binding site of the MamE PDZ domain lies between α1 and β3 and contains a large number of hydrophobic amino acids, similar to the HtrA2-PDZ domains fold which is known to be a binding site for single peptide ligands or hydrophobic amino acids of other proteins (Zhang et al., 2007; Wang et al., 2010). Prediction of the electrostatic density map displays negative and positive patches on the PDZ surface, which is similar to other HtrA2 PDZ domains. Based on all the previous results, we can assume that MamE acts as a serine protease and that its PDZ domain can interact with other proteins that are involved in magnetosome formation.

### MamH

**FIGURE 5:**
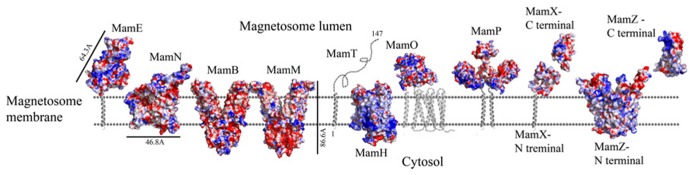
**Protein structure predictions that are involved in iron transport and nucleation.** Negative charges are in red and positive charges are in blue. Black bars define the protein size in angstroms. Magnetotactic ovoidal bacterium MO-1 MamP structure from was magnetotactic ovoidal bacterium MO-1 is determent by X-ray crystallography.

MamH is a 428 amino acid protein and in most MTB it is located in the *mamAB* operon, though its function and how it is involved in magnetosome formation are both unknown (Murat et al., 2010; Lohsse et al., 2011). In MSR-1, MamH is highly similar to MamZ (Raschdorf et al., 2013). Deletion of MamH results in a decrease in the number and size of magnetosomes (Murat et al., 2010; Raschdorf et al., 2013). It was shown that *mamH* deletion in *ΔmamZ* mutants causes a strong effect on the crystals’ size, shape, and magnetic response (Raschdorf et al., 2013). Therefore, it was suggested that MamH may be involved in magnetite biomineralization (Raschdorf et al., 2013). By searching for homologous proteins or conserved domains with the BLAST server we found similarities to a conserved domain in the Major Facilitator Superfamily (MFS), members of which are known to function as membrane transporters (Pao et al., 1998; Marchler-Bauer et al., 2011; Raschdorf et al., 2013). MFS proteins are single-polypeptide secondary carriers that use the electrochemical potential of the transported substrates (Pao et al., 1998). Prediction of MamH secondary structure shows an organization of 12 transmembrane helices with short connecting loops and a longer loop connecting H6–H7 (Raschdorf et al., 2013; **Figure 3**). This organization is simlar to MFS protein structures (Huang et al., 2003). The MamH predicted structural model displays a negative cavity which can bind positive ions or ligands like iron and transfer them through the magnetosome membrane (PDB ID: 2XUT). The hypothesis that MamH is an integral membrane protein cannot only be based on the sequence prediction but also on the model’s electrostatic density map. This contains the positive girdle envelope around the protein exterior, which is usually a characteristic feature of integral membrane proteins (**Figures 3** and **5**). Another structure of MFS proteins indicates a proton-coupled transporter which is essential for phosphate uptake in plants and fungi. The *Piriformospora indica* phosphate transporter (PiPT) is a high affinity phosphate transporter that is involved in improving phosphate nutrition levels in the host plant and is known to be a phosphate/H^+^ symporter from the MFS (Pao et al., 1998; Pedersen et al., 2013). By using Swiss-PdbViewer we created manually another MamH model structure based on PiPT structure which provided a different view for MamH function (PDB ID: 4J05; Guex and Peitsch, 1997). In the MamH PiPT-based electrostatic map we can detect a positive pocket in the magnetosomal side and a negative patch in the cytosolic side. These distinctions are similar to PiPT structure and may result in a new perspective on MamH function. Recently, it was shown in *M. gryphiswaldense* MSR-1 that there are two iron phases during the biomineralization process: ferrihydrite with high phosphorus content – similar to the bacterial ferritin core – and magnetite (Fdez-Gubieda et al., 2013). It has also been suggested by Baumgartner et al. (2013) that magnetite formation starts from a phosphate-rich ferric hydroxide phase, through a short-lived ferrihydrite phase followed by a final phase of magnetite. These results support our MamH model, which can function as a phosphate transporter clearing phosphate from the magnetosome while biomineralizing magnetite (Fdez-Gubieda et al., 2013).

### MamN

MamN is a 437 amino acid protein and its deletion leads to an empty magnetosome chain phenotype (Murat et al., 2010). The inability to synthesize magnetite within the magnetosomes can indicate MamN involvment in biomineralization, iron transport, magnetite nucleation, or chemical environment determination needed for magnetite synthesis in the magnetosome (Murat et al., 2010). However, its deletion does not affect the localization of other magnetosome proteins (Murat et al., 2010).

MamN has homology to a Na^+^/H^+^ antiporter; this activity can influence the pH within the magnetosome core (Komeili, 2012). It is known that magnetite synthesis requires a basic environment which raises the speculation that a H^+^ extruder is needed (Komeili, 2012).

Prediction of the MamN secondary structure, using XtalPred server, presents 11 integral membrane helices (Slabinski et al., 2007). The MamN 3D model structure is based on a sodium-dependent dicarboxylate transporter (NaDC) template (**Figure 3**; PDB ID: 4F35; Arnold et al., 2006). NaDC is a plasma membrane protein which transports tricarboxylates or dicarboxylates and is known to bind a specific substrate (Mancusso et al., 2012). NaDC contains 11 transmembrane helices and forms a dimer to create a large interface area (Mancusso et al., 2012). MamN model structure shows membrane protein characteristics such as the uncharged girdle and the charged core (**Figure 5**). The MamN model fits only to the monomeric NaDC but not to the dimeric NaDC structure. Further attemps to create a 3D structure of MamN based on its Na^+^/H^+^ antiporter homologous protein did not result in a convincing structure.

### MamM and MamB

MamM and MamB are large proteins – 34.4 and 31.9 kDa, respectively – and are magnetosome membrane proteins which may be involved in iron transport due to their similarity to the cation diffusion facilitator (CDF) protein family (Uebe et al., 2011). CDF proteins are found in all kingdoms of life and are involved in the transport of divalent metal cations (Paulsen and Saier, 1997). Most members of the CDF family contain six highly conserved transmembrane helices, organized as a transmembrane domain, with cytoplasmic N- and C-terminals (Cragg et al., 2002). It was shown that CDFs create a homodimeric structure and use a proton antiport mechanism to drive substrate translocation across the lipid membrane (Haney et al., 2005; Lu et al., 2009). MamM deletion causes the loss of magnetite crystal formation and results in empty magnetosomes (Uebe et al., 2011). Except for its suggested role in iron transport, MamM is also involved in crystallization initiation and proper localization of other magnetosome proteins (Uebe et al., 2011). In contrast to MamM, MamB deletion causes a lack of magnetosome vesicles (Murat et al., 2010; Uebe et al., 2011). Further experiments indicated that MamM and MamB can interact, and that MamM is required for MamB stabilization (Uebe et al., 2011). It has also been shown that the cytoplasmic CTD in MamM and MamB are involved in MamB stabilization (Uebe et al., 2011)

Replacement of the conserved cysteine residues (Cys9/138) with serine or alanine did not affect MamM function but similar mutations in MamB abolished its function (Uebe et al., 2011). Mutation of Cys138 in MamB blocked its oligomerization and magnetosome formation (Uebe et al., 2011). In MSR-1 the MamB C-terminal has a TPR recognition signature and was shown to interact with MamE’s PDZ domain (Quinlan et al., 2011; Uebe et al., 2011). In addition, point mutations in the MamM membranal putative metal binding site (Y46H, Y46D, D50A, H155A, and D159A) lead to changes in crystal size and morphology, which may be the result of a reduction in iron transport rates into the magnetosome vesicles or of the nucleation of magnetite crystals (Uebe et al., 2011). It seems that magnetite can only be stable in a pH range from ~7 to 14 (Bell et al., 1987). Therefore, if MamM is using a H^+^/cation antiport mechanism similar to other CDF proteins, it may explain why dysfunction of MamM causes defects in crystal formation (Uebe et al., 2011)

MamM and MamB secondary structure prediction indicates a transmembrane domain with six α-helices followed by a C-terminal cytosolic domain (Uebe et al., 2011). MamM and MamB 3D structures prediction is based on the FieF structure and built manually in SPDB viewer (PDB ID: 3H90; **Figure 3**; Guex and Peitsch, 1997; Uebe et al., 2011). FieF is an *E. coli*, homodimeric, zinc transporter (Lu et al., 2009). Its CTD has a metallochaperone-like fold which is found in cytoplasmic metal carrier proteins (O’Halloran and Culotta, 2000). The FieF active sites which participate in zinc transport are located toward the center of each transmembrane domain and continue to the CTD–CTD interface (Lu et al., 2009). In FieF structure there are four amino acids that bind a zinc ion in a negative pocket located between helix 2 and helix 5 (Asp45, Asp49, His153, and Asp157; Lu et al., 2009). By overlapping the MamM model with FieF’s structure we can observe similar amino acids at the zinc binding positions (**Figure 4B**). These amino acids were mutated in MamM leading to protein dysfunction *in vivo*. Similar zinc binding site positions can be found when we overlap MamB onto FieF. There are four residues in MamB that may act as an ion-binding site: His46, Asp50, Asp154, and Asp158 (**Figure 4C**). From multiple sequence alignment using the ClustalW server, we can detect that there are no amino acid conservations between MamM, MamB and FieF CTDs sequences [5]. In MamM CTD we found that the amino acids that are involved in zinc binding in FieF are different and create a negative pocket that may be involved in ion binding. We cannot find such a negative pocket in the MSR-1 MamB 3D model structure that may indicate different activity for MamB (**Figure 5**).

### MamP

MamP is a 270 amino acid protein that is predicted to have one transmembrane helix on its N-terminal and two cytochrome c-like motifs (CXXCH) on its C-terminal, similar to MamE and MamT proteins (Siponen et al., 2012). MamP also contains a PDZ domain signature and a putative signal sequence that suggests it is targeted to the inner membrane (Siponen et al., 2012). Mutation in MamP from *M. magneticum* AMB-1 strain causes deficiencies in crystal maturation and the loss of the magnetic response (Siponen et al., 2012). It was suggested that in *M. magneticum* AMB-1 strain MamP could play a role in controlling crystal number and size (Murat et al., 2010). Furthermore, MamP can be involved in the electron transfer chain which is important for magnetosome assembly and magnetite formation (Siponen et al., 2012).

Recently, MamP structure (residues 26–260) from *Magnetotactic ovoidal bacterium* MO-1 strain was determined (Siponen et al., 2013). MamP 3D structure presents a PDZ fold followed by two magnetochrome domains with a 17 residues linker between them (PDB ID: 4JJ0). It was shown that MamP PDZ domain has different properties from other known PDZ domains which are known to interact with peptides from other proteins and may be involved in the protein dimerization state (Iwanczyk et al., 2007). MamP PDZ domains are highly conserved in other species which indicate conservation of the protein dimeric state (Siponen et al., 2013). In *M. gryphiswaldense* MSR-1 MamP structure 3D model, which is based on the determined MamP structure, show similar characterization (**Figure 3**). It was shown that MamP structure has a highly negative pocket between the two monomers that includes conserved acidic residues (Glu91, His93, Glu98, Glu123, and Glu193) that were suggested to act as an iron-binding site (**Figure 5**). Mutation in these acidic residues to alanine *in vivo* led to magnetic response and crystal size defects (Siponen et al., 2013).

### MamT

In *M. gryphiswaldense* MSR-1, MamT contains 174 amino acids and is predicted to have a double cytochrome c CXXCH motif and a membrane-anchoring hydrophobic helix on its N-terminal (Slabinski et al., 2007). MamT secondary structure prediction shows that the CTD is located on the magnetosome lumen and include α-helices and β-sheets (**Figure 3**; Slabinski et al., 2007). A *ΔmamT* mutant shows defects in crystal maturation and the loss of magnetic response (Murat et al., 2010; Siponen et al., 2012).

### MamZ

MamZ is a 661 amino acid protein and is known to have similarities to the ferric reductase-like transmembrane component (YedZ-like domain) on its C-terminal side, whilst its N-terminal has homology to the MFS (Richter et al., 2007; Raschdorf et al., 2013). Recently it was shown that MamZ interacts with the magnetosome membrane and was suggested to create an iron oxidoreductase and transport complex with MamX and MamH (Raschdorf et al., 2013). MamZ deletion shows a slightly reduced magnetic response with two types of crystals that are still aligned into a chain (Raschdorf et al., 2013). Deletion of MamZ C-terminal, which shares homology with YedZ-like domain, reveals the same phenotype as the full MamZ deletion, suggesting that this domain has a critical role in the biomineralization process (Raschdorf et al., 2013).

Prediction of MamZ 3D structure is based on two different templates. The N-terminal is predicted based on the glycerol-3-phosphate transporter (GlpT) from *E. coli,* which belongs to the MFS transporter family (PDB ID: 1PW4; Kelley and Sternberg, 2009). GlpT is known to transport glycerol-3-phosphate into the cytoplasm and inorganic phosphate to the periplasm and is composed of 12 transmembrane helices (Huang et al., 2003). MamZ N-terminal model structure presents 12 transmembrane helices with a negative pore facing the magnetosomal side, similar to GlpT (**Figure 5**). The electrostatic density map of MamZ cytosolic side is different from GlpT which is highly positively charged and may indicate a different function (**Figure 5**; Huang et al., 2003). MamZ C-terminal is predicted to have a domain structure from cytochrome bc_1_ complex, which is known as ubiquinol-cytochrome c reductase (PDB ID: 3CX5; **Figure 3**; Kelley and Sternberg, 2009). Cytochrome c’s function is to transfer electrons from the cytochrome bc_1_ complex to cytochrome c oxidase (Solmaz and Hunte, 2008). MamZ C-terminal model structure includes three α-helices and two short β-sheets. The electrostatic density map of MamZ presents two highly charged patches – positive and negative – that are predicted to face the magnetosome lumen (**Figures 3** and **5**).

### MamX

The *mamX* gene is conserved in most magnetite-producing α-proteobacteria (Abreu et al., 2011). It has a weak similarity to both the magnetosome serine-like protease MamE and to MamS (Richter et al., 2007; Lohsse et al., 2011). Structure prediction of MamX presents a transmembrane helix on its N-terminal side, two cytochrome c-like domains and a DNA-binding domain on its C-terminal (Söding, 2005; Slabinski et al., 2007). As mentioned above, cytochrome c functions as an electron carrier between different redox partners and can also be found in MamP, MamE, and MamT (Siponen et al., 2012). Deletion of these three proteins shows defects in crystal maturation (Siponen et al., 2012). It was shown that deletion of the cytochrome c domain in MamX abolishes its function (Raschdorf et al., 2013). The two cytochrome c-like domains are predicted to be located between Tyr42 and Val111 and their structures were predicted based on four different templates of cytochrome c domains (PDB ID: 1OGY_B, 1JNI_A, 3ML1_B, and 1QO8_A; **Figure 3**; Söding, 2005). In all these templates the cytochrome c domain creates a ring-like shape around the heme groups, except for the MamX cytochrome c-like domain prediction. By using HHpred server the C-terminal is predicted to be shaped like an OB fold motif (PDB ID: 2CQA_A, 3KDF_D, 3KF6_A; **Figure 3**; Söding, 2005). This motif is observed as a binding domain for oligonucleotides or oligosaccharides (Murzin, 1993). The OB-fold has a five β-sheet coiled fold that forms a closed β-barrel with an α-helix located between the third and fourth strands (Murzin, 1993). The MamX C-terminal predicted structure contains three β-strands followed by an α-helix, similar to the OB fold (Söding, 2005).

## CRYSTAL SHAPE AND SIZE

### MamR

MamR was shown to be important for crystal number and size control but is not involved in the control of their morphology (Murat et al., 2010; Quinlan et al., 2011). MamR is a small, ~72 amino acid protein with a predicted DNA-binding domain similar to the HTH-17 superfamily (Marchler-Bauer et al., 2011). MamR is also predicted to have a DNA-binding domain similar to the excisionase (Xis) family (Marchler-Bauer et al., 2011). Phage-encoded excisionase (Xis) proteins are involved in DNA architectural structure and are needed for recruiting integrase to a specific binding site for its excision (Sam et al., 2002). MamR secondary structure prediction shows a fold of four α-helices and two short β-strands (Slabinski et al., 2007).

**FIGURE 6:**
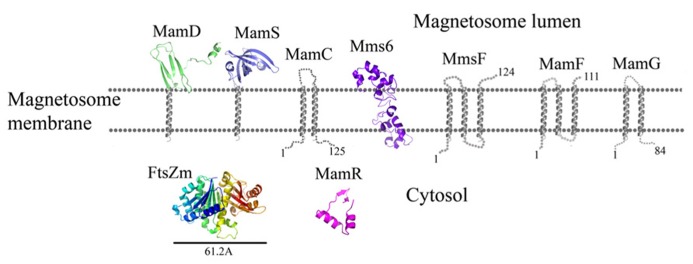
**Protein structure predictions that are involved in controlling the size and shape of the magnetite particles.** Structures are in ribbon representation. Predictions of anchoring transmembrane helices are in gray. Black bars define the protein size in angstroms.

**FIGURE 7:**
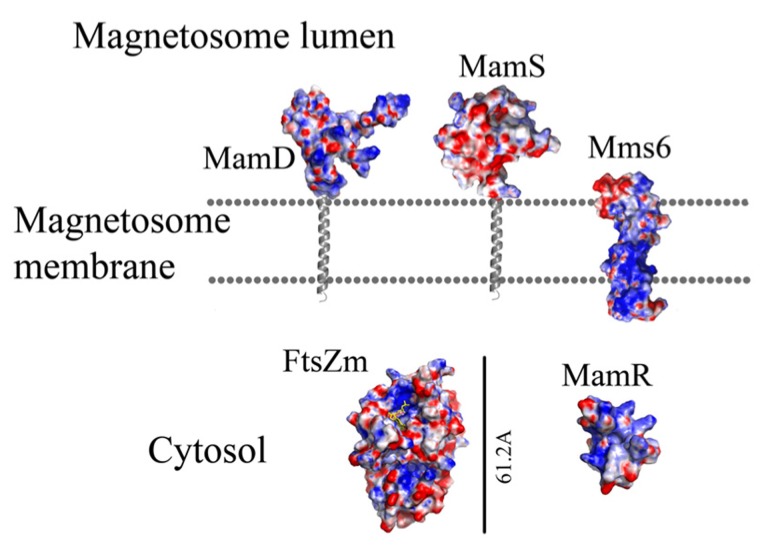
**Protein structure predictions that are involved in controlling the size and shape of the magnetite particles.** Negative charges are in red and positive charges are in blue. Predictions of anchoring transmembrane helices are in gray. Black bars define the protein size in angstroms.

MamR model structure is based on the DNA-binding protein Rv2175c, from *M. tuberculosis* (PDB ID: 2KFS; Cohen-Gonsaud et al., 2009). Rv2175c is activated by phosphorylation of its N-terminal domain (Cohen-Gonsaud et al., 2009). The MamR model presents a C-terminal with two α-helices followed by two β-sheets and a negative patch on the protein surfaces (**Figures 6** and **7**; Kelley and Sternberg, 2009).

### MamS

MamS is a ~19 kDa protein and its deletion leads to a weak magnetic response due to defects in magnetite crystal size and morphology (Murat et al., 2010). In *M. magneticum* AMB-1 strain several small magnetic particles were clustered together in one magnetosome membrane, suggesting MamS involvement in the magnetite post-nucleation event (Murat et al., 2010; Komeili, 2012). According to BLAST server there is no conserved domain in the MamS sequence (Marchler-Bauer et al., 2011). Prediction of its secondary structure indicates a fold containing a transmembrane helix on the N-terminal followed by β-strands on the cytoplasmic side (**Figure 6**; Slabinski et al., 2007). Prediction of the MamS 3D structure is based on the structure of the hypothetical protein YgiW from *E. coli* with unknown function (PDB ID: 1NNX; Kelley and Sternberg, 2009). The MamS structure model displays six β-sheets with two short α-helices, similar to the OB fold which is known to create a β-barrel structure that can bind oligonucleotides or oligosaccharides (Murzin, 1993).

### FtsZ-LIKE

The *ftsZ*-like gene was found to be a second copy of the gene encoding the tubulin-like FtsZ protein in *Magnetospirillum* species, but missing the 200 amino acid C-terminal tail (Ding et al., 2010). Deletion of *ftsZ*-like does not affect cell division but results in smaller magnetite crystals (Ding et al., 2010). It was shown that FtsZ-like displays both ATPase and GTPase activities and also has GTP-dependent polymerization into filaments *in vitro* (Ding et al., 2010). FtsZ proteins are known to be involved in cell division and create the septum which allows the cell to divide into two daughter cells (Scheffers and Driessen, 2001). FtsZ is known to have GTPase activity and to create a filamentous structure within the cell (Mukherjee and Lutkenhaus, 1998). The FtsZ 3D structure contains two domains, one of which is the N-terminal domain, which has a Rossman fold containing the GTP binding site and the other is the CTD, which is required for proper polymerization of FtsZ and interaction with other proteins (Din et al., 1998; Löwe and Amos, 1998).

Prediction of the FtsZ-like 3D structure shows high similarity to other FtsZ proteins (**Figure 6**; PDB ID: 2RHL; Arnold et al., 2006). The FtsZ-like structure model presents the two known FtsZ protein domains. The electrostatic density map fits the GTP binding pocket characteristics (**Figure 7**). It can be assumed from the structure and the high homology between the FtsZ proteins that FtsZ-like may be involved in cell division processes (Altschul et al., 1990).

### MamC

MamC (also known as Mms13) is a small, 12.4 kDa protein and is strongly conserved in most magnetotactic α-proteobacteria (Abreu et al., 2011). Deletion of MamC does not present any defects in crystal size or shape and is tightly associated with the magnetosome membrane in *M. gryphiswaldense* MSR-1 strain (Wawer et al., 2001; Scheffel et al., 2008). Δ*mamE* cells show mislocalization of MamC-GFP to the magnetosome chain (Quinlan et al., 2011). Prediction of MamC secondary structure with XtalPred (Slabinski et al., 2007) displays two transmembrane helices with a connecting loop that is predicted to interact with the magnetite crystal (**Figure 6**). Based on the secondary structure prediction, the MamC connecting loop is predicted to adopt an α-helix structure with several charged residues.

### MamG

MamG is a small, 8 kDa protein specifically expressed in the magnetosome membrane (Lang and Schüler, 2008). MamG has no homologous proteins and contains a similar Leu-Gly dipeptide motif to MamD and Mms6 (Scheffel et al., 2008). Prediction of its secondary structure in the XtalPred server displays two transmembrane helices and an unstructured C-terminal that faces the bacteria cytosol (**Figure 6**). Sequence analyses indicate that most of the protein’s amino acids are hydrophobic except for the last residues, which are negatively (Asp68, Glu78, and Glu82) and positively charged (Lys71, Arg73, and Lys74).

### MamD

MamD is a 30.2 kDa protein which contains a transmembrane helix on its C-terminal and a Leu-Gly repeat domain on its N-terminal, which is located in the magnetosome lumen (Richter et al., 2007; Arakaki et al., 2008). MamD is one of the magnetosome membrane-associated proteins which controls magnetite crystal size (Scheffel et al., 2008). Its secondary structure prediction shows that the N-terminal is located within the magnetosome lumen with β-sheets and α-helices. Prediction of the MamD 3D structure shows only a small part of the magnetosomal side (Gly116 to ALA 158) and displays a β-sheets fold which is based on the CTD of FlgD protein from *Xanthomonas campestris* (PDB ID: 3C12; **Figure 6**; Kelley and Sternberg, 2009). FlgD is a scaffold protein and is required for flagellar hook assembly (Ohnishi et al., 1994). Sequence alignment between MamD and FlgD presents low sequence identity, which does not suggest a function for MamD. Most of the residues in the MamD structure are hydrophobic with a few polar or negatively charged residues (Kelley and Sternberg, 2009).

### MamF

MamF is a small, 12.3 kDa protein which shares 61% identity with MmsF (Lohsse et al., 2011). MamF was shown to be protected against proteolytic degradation due to its integral membrane localization and its highly hydrophobic nature with three predicted transmembrane helices (Gru et al., 2004). *In vivo* localization of MamF-GFP was identified as linear spots along the cell axis in accordance with the magnetosome chain localization (Lang and Schüler, 2008). MamF secondary structure prediction shows three transmembrane helices with short connecting loops (**Figure 6**; Kelley and Sternberg, 2009). The first loop which predicted to be located in the magnetosomal side and includes highly charged residues (Arg39, Asp40, Asp41, and Glu42) which may indicate a possible interaction with the magnetite particles (McGuffin et al., 2000; Kelley and Sternberg, 2009).

### Mms6

In *M. gryphiswaldense* MSR-1 strain, Mms6 is a small, 136 amino acid protein that was suggested to undergo proteolytic cleavage from its proprotein (Gru et al., 2004). In *M. magneticum* AMB-1 strain, Δ*mms6* strains showed smaller magnetite crystals with different shapes (Tanaka et al., 2011; Murat et al., 2012). The Mms6 C-terminal is highly acidic and the region between the middle and the C-terminal contains basic amino acids (Arakaki et al., 2003). It was shown that Mms6 possesses iron-binding activity and it was suggested that the C-terminal region can initiate crystal nucleation during magnetite formation and direct the shape of magnetite crystals *in vitro* (Arakaki et al., 2003). Mms6, MamD and MamG have a common Leu and Gly repetitive sequence (Arakaki et al., 2008). Mms6 can self-assemble into micelles *in vitro* by interactions between the cleaved Mms6 N- and CTDs and, due to iron binding, a conformational change is induced (Feng et al., 2013).

Prediction of the Mms6 secondary structure shows an N-terminal domain that is predicted to be unstructured followed by one transmembrane helix and a C-terminal, which may form an α-helix structure (**Figure 6**). Mms6 model structure was predicted in 3Dpro server which use structural characterization and statistical terms in the energy function (Cheng et al., 2005).The electrostatic density map of Mms6 3D structure model results in a negative patch on its CTD that can act as an iron binding site (**Figure 7**). Analysis of the Mms6 model structure and protein sequence indicates that the predicted transmembrane helix (G91 to Y115) contains only hydrophobic residues, which may support the existence of such a helix.

### MmsF

MmsF is a 124 amino acid protein and is predicted to have three transmembrane helices but does not show any conserved domain (Slabinski et al., 2007; Murat et al., 2012;). In the absence of *mmsF* in *M. magneticum* AMB-1 strains, magnetite synthesis initiation is not affected but the crystal growth is stalled (Murat et al., 2012). It was found that the MmsF sequence is highly homologous between *M. gryphiswaldense* MSR-1 and *M. magneticum* AMB-1 (Murat et al., 2012). MmsF is shown to control crystal size and shape in *M. magneticum* AMB-1 cells (Murat et al., 2012). MmsF was also shown to be associated with the magnetosome membrane by fusing a GFP tag to its N-terminal (Murat et al., 2012). The MmsF N-terminal is located in the cytoplasmic side whilst the C-terminal faces the magnetite crystals (**Figure 6**; Murat et al., 2012).

## CONCLUSION

**FIGURE 8:**
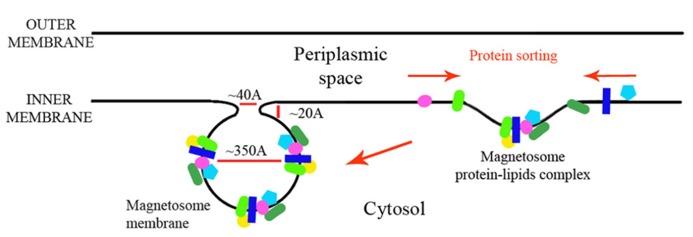
**Magnetosome membrane invagination model**.

In this paper we looked at the MAPs, which are encoded in the *M. gryphiswaldense* MSR-1 MAI region. We analyzed each protein sequence and created structural models that will enable better understanding of their function in magnetosome formation (**Table 1**). By analyzing the size of these proteins some key questions regarding the magnetosome membrane invagination can be answered. One of the questions that still exist in the magnetosome field regards how the magnetosome membrane invaginates. In the literature, the invagination process is described as a curving of the inner membrane that creates a pocket shape. Yet, neither the driving force nor the player were determined in MTB (Komeili et al., 2006). One possible option is that the inner membrane starts to fold into a vesicle – by a yet unknown mechanism – followed by the localization of magnetosome proteins to the vesicles. However, the diameter of the invagination membranous neck is only about 40 Å or less (Komeili et al., 2006) with a highly curved concave turn followed by a convex membrane structure (**Figure 8**). This geometry is very limited in size and shape, especially with the concave turn. Based on our models, many proteins have domains that are larger in size than the magnetosome invagination diameter. For example, MamB, which is essential to the magnetosome invagination, has a cytoplasmic domain with a size of ~52 Å and a magnetosomal domain in the size of ~37 Å (**Figure 3**). Other examples are MamO and MamE, both with large, CTD (~36 Å in size; **Figure 3**). Even by looking at these few examples we can determine that such proteins cannot pass through this magnetosome invagination neck due to their size, especially if this part is held by other proteins, as suggested by Tanaka et al. (2010). A more probable mechanism that fits our structural data is that the magnetosome proteins are sorted prior to the magnetosome invagination and accumulate on the inner membrane as protein–protein–lipid complexes that might be mimicking lipid rafts (**Figure 8**). The formation of such a complex, together with the natural curvature of the integral magnetosome membrane helices, may lead to a natural invagination without a special protein support; a process that will gain more force as more proteins are targeted into the membrane complex. Based on the genomic data, deletion of several MAPs (MamB, MamQ, MamI, and MamL) leads to the abolishment of magnetosome invagination (Murat et al., 2010) and we can suggest these as hubs for the protein–protein interaction, or as proteins with correct curvatures needed for the magnetosome invagination.

**Table 1:** Summary and characteristic of all magnetosome-associated proteins that are encoded in *Magnetospirillum gryphiswaldense* MSR-1strain.

Protein	Number of amino acid	Transmembrane helices	Domain	Predicted structure template	% Structure confidence or sequence identity (*E* value)	Major function in
MamE	591	1 (27–48)	Magnetochrome, trypsin and PDZ	2Z9I	100%	Biomineralization
MamT	174	1 (10–28)	Magnetochrome	Secondary structure only	–	Biomineralization
MamO	632	8 (1-N-terminal, 7-C-terminal)	Trypsin and anion transport	1LCY	17%	Biomineralization, iron transport
MamH	428	12	MFS, similar to MamZ	2XUT, 4JOD (PiPT)	100%	Biomineralization
MamI	77	2	No conserved domain	Secondary structure only	–	Membrane invagination
MamP	270	1 (N-terminal)	PDZ, magnetochrome	4JJ3 (MamP, MO-1)	Structure was determent	Biomineralization
MamN	437	11	Na^+^/H^+^ antiporter	4F35 (monomer)	100%	Biomineralization, iron transport and nucleation
MamD	314	1 (C-terminal)	No conserved domain	3C12 (FlgD) – only N-terminal	77.10%	Crystal shape
MamM	318	3	CDF, metal carrier	3H90 (FieF)	100%	Iron transport
MamB	297	3	CDF, metal carrier	3H90 (FieF)	100%	Iron transport
MamQ	273	1 (N-terminal)	LemA protein	2ETD (LemA)	100%	MM formation
MamR	72	–	DNA-binding	2KFS (RV2175c)	95.50%	Crystal shape
MamS	180	1 (N-terminal)	OB fold	1NNX (YgiW)	91.80%	Post-nucleation
MamZ	661	18	YedZ- like domain (C-terminal), MFS (N-TER)	1PW4 (GlpT), 3CX5 (cytochrome C)	N-terminal (100%), C-terminal (32.86%)	Biomineralization (C-terminal)
MamJ	426	–	CAR domain mostly unstructured, TonB	Unstructured protein	–	Magnetosome arragment into chain structure
						
MamK	360		Filaments structure (MreB, ParM)-actin-like, ATP binding site	1JCF (MreB)	100%	Magnetosome arragment into chain structure
						
MamL	123	2	Similar to MamI	Secondary structure only	–	Membrane invagination
MamA	217	–	TPR	3AS4 (MamA, AMB-1), 3AS8 (MamA, MSR-1), 2MUC	Structure was determent	Surrounded the magnetosome vesicles
				(MamA, *M. bavaricum*)		
MamU	297	–	DGKs	3T5P	2.00E-15	Membrane invagination
MamX	269	1 (N-terminal)	Similar to MamE and MamS, cytochrome, DNA-binding domain	1OGY, 1JNI, 3ML1, 1QO8 (cytochrome C) 2CQA, 3KDF, 3KF6 (OB fold)	90% (N-terminal)	Crystal maturation
					69% (C-terminal)	
MamY	371	2 (N-terminal)	BAR domain	1SJ8 (talin)	95.40%	Membrane invagination
FtsZ like	323	–	GTPase actin domain	2RHL	1.00E-92	Crystal size and cell division
MamC	125	2	No conserved domain	Secondary structure only	–	Crystal size and shape
MamG	84	2	Similar to MamD and Mms6	Secondary structure only	–	Crystal size and shape
MamF	124	3	Similar to MmsF	Secondary structure only	–	Crystal size and shape
Mms6	136	1	C-terminal highly acidic involved in nucleation	Base on energy calculations of the sequence		Crystal size and shape

From the prediction of structures it can also be shown that some of the proteins have protein–protein interaction domains (such as PDZ and TPR domains). Such domains can be indicative of the creation of possible protein networks that are involved in magnetosome formation. It has already been shown that such interactions exist between MAPs; for example, between the MamE PDZ domain and the CTD of MamB (Uebe et al., 2011), between MamK and MamJ, and even between MamA and other proteins (Yamamoto et al., 2010). Except for the protein–protein interaction domains there are other redundant predicted domains that appear in magnetosome proteins such as: proteases (MamO and MamE), cytochrome c domains (MamE, MamT, MamX, and MamZ), CDF (MamM and MamB), and transporters (MamO, MamN, MamH, and MamZ). This redundancy can be found in the function of all four genes in the *mamCD* and *mms6* operons which control the size and shape of magnetite crystals (Scheffel et al., 2008). These repetitive functions and domains indicate the ability of the bacteria to ensure magnetosome formation and its activity.

Another question that is raised from protein structure predictions and protein orientations in the magnetosome membrane is why we do not see a gap between the magnetosome membranes and the mineral crystal in electron microscopy (EM) images. In EM experiments of magnetosome vesicles the magnetosome membrane is attached directly to the magnetic particle without a ~3 nm gap that will fit our predicted protein domain (Murat et al., 2010; Abreu et al., 2013). The distance between the magnetosome membrane and the particle is not enough to contain the proteins that are directed to the magnetosome lumen (MamQ, MamE, MamO, MamP, MamX, MamD, and MamZ; **Figures 1**, **3** and **6**). One explanation is that this may be the result of a similar density between the membrane and proteins together with a large contrast between the mineral and the membrane that hinders the detection protein gaps, as seen in EM. Another hypothesis is that protein degradation by magnetosome proteases is taking place during mineral maturation that reduces the proteins’ outer membrane domains. Yet, more experiments will need to be conducted in order to resolve this issue.

## METHODS

All MTB protein sequences were extracted from the NCBI website^2^, followed by homologous protein and conserved domain searching with the BLAST server^3^. To analyze the protein sequences and to predict their structures we used several different servers: XtalPred^4^ and PsiPred^5^ were used to identify protein secondary structure (McGuffin et al., 2000; Slabinski et al., 2007). Swiss-Model^6^, HHpred^7^, Phyre^8^, and PsiPred servers were used to predict the protein structures and to identify their possible functions (Guex and Peitsch, 1997; McGuffin et al., 2000; Söding, 2005; Kelley and Sternberg, 2009). Each model structure was energetically minimized in the Swiss PDB Viewer program for a few energy minimization cycles (Guex and Peitsch, 1997). Electrostatic calculations were performed via the APBS tool and images were prepared using PyMOL (Baker et al., 2001; DeLano, 2002). All the obtained model structure files are available at .

## Conflict of Interest Statement

The authors declare that the research was conducted in the absence of any commercial or financial relationships that could be construed as a potential conflict of interest.

## Footnotes

1

2

3

4

5

6

7

8
